# Disturbed flow–induced G_s_-mediated signaling protects against endothelial inflammation and atherosclerosis

**DOI:** 10.1172/jci.insight.140485

**Published:** 2020-12-03

**Authors:** Akiko Nakayama, Julián Albarrán-Juárez, Guozheng Liang, Kenneth Anthony Roquid, András Iring, Sarah Tonack, Min Chen, Oliver J. Müller, Lee S. Weinstein, Stefan Offermanns

**Affiliations:** 1 Max Planck Institute for Heart and Lung Research, Department of Pharmacology, Bad Nauheim, Germany.; 2 Metabolic Diseases Branch, National Institute of Diabetes and Digestive and Kidney Diseases, NIH, Bethesda, Maryland, USA.; 3 Department of Internal Medicine III, University of Kiel, Kiel, and German Center for Cardiovascular Research (DZHK), Partner site Hamburg/Kiel/Lübeck, Germany.; 4 Centre for Molecular Medicine, Medical Faculty, J.W. Goethe University Frankfurt, Frankfurt, Germany.; 5 DZHK RheinMain, Germany.

**Keywords:** Inflammation, Vascular Biology, Atherosclerosis, G-proteins

## Abstract

Atherosclerosis develops preferentially in areas of the arterial system, in which blood flow is disturbed. Exposure of endothelial cells to disturbed flow has been shown to induce inflammatory signaling, including NF-κB activation, which leads to the expression of leukocyte adhesion molecules and chemokines. Here, we show that disturbed flow promotes the release of adrenomedullin from endothelial cells, which in turn activates its G_s_-coupled receptor calcitonin receptor–like receptor (CALCRL). This induces antiinflammatory signaling through cAMP and PKA, and it results in reduced endothelial inflammation in vitro and in vivo. Suppression of endothelial expression of Gα_s_, the α subunit of the G-protein G_s_; CALCRL; or adrenomedullin leads to increased disturbed flow–induced inflammatory signaling in vitro and in vivo. Furthermore, mice with induced endothelial-specific deficiency of Gα_s_, CALCRL, or adrenomedullin show increased atherosclerotic lesions. Our data identify an antiinflammatory signaling pathway in endothelial cells stimulated by disturbed flow and suggest activation of the endothelial adrenomedullin/CALCRL/G_s_ system as a promising approach to inhibit progression of atherosclerosis.

## Abstract 2

Disturbed flow promotes the release of adrenomedullin from endothelial cells and activates Gs-mediated signaling, which reduces endothelial inflammation in vitro and in vivo.

## Graphical abstract

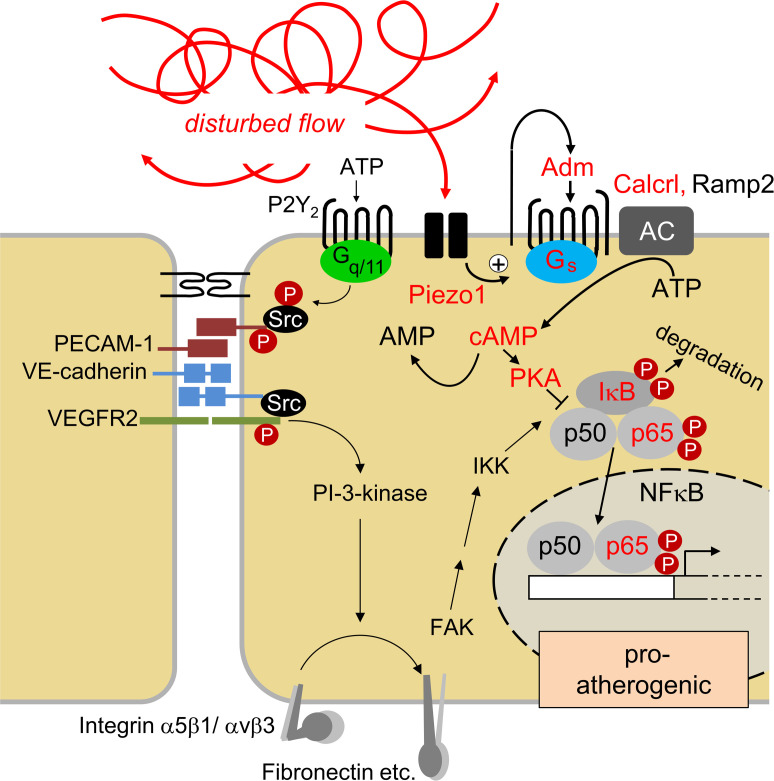

## Introduction

Atherosclerosis predisposes to myocardial infarction and stroke, which are leading causes of morbidity and mortality (1). It is an inflammatory disorder of large- and medium-sized arteries, which is promoted by a variety of risk factors, including arterial hypertension, high plasma levels of LDL-cholesterol and triglycerides, diabetes mellitus, obesity, and sedentary life style (1–3). In addition to these systemic factors, the local microenvironment strongly affects the development and progression of atherosclerosis, which preferentially develops in areas of disturbed blood flow such as branching points or curvatures of the arterial system (4–6). Work of the last decades has identified several endothelial signaling pathways and downstream events that are induced by disturbed flow, but not by laminar flow, and that promote proatherogenic phenotypes (4–12). A major inflammatory signaling pathway induced by disturbed flow is the activation of NF-κB — which, in turn, leads to the expression of inflammatory mediators, such as the chemokine CCL2 and of leukocyte adhesion molecules, including VCAM-1 and E-selectin (13–15). Activation of inflammatory signaling by disturbed flow involves a mechanosignaling complex consisting of PECAM-1, VE-cadherin, and VEGFR2 (6, 16), as well as activation of integrins — in particular, integrin α5 (17–19). Activation of integrins can induce NF-κB activation through focal adhesion kinase (FAK) (20–23). Activation of integrin α5, in addition, results in inhibition of cAMP signaling through the activation of phosphodiesterase 4D (PDE-4D) (18).

Previous study showed that disturbed flow activates the mechanosensitive cation channel PIEZO1, which induces activation of the purinergic P2Y_2_ receptor and G_q_/G_11_-mediated signaling and mediates disturbed flow–induced activation of the mechanosignaling complex and of integrin α5β1 (24). While laminar flow induces the same upstream signaling cascade involving PIEZO1, G_q_/G_11_-mediated signaling, and the mechanosignaling complex, it does not induce the integrin-dependent NF-κB activation when applied for longer time periods; rather, it leads to AKT-dependent eNOS phosphorylation and activation (24). We have recently shown that laminar flow also activates PIEZO1, which leads to the release of adrenomedullin from endothelial cells and subsequent activation of its G_s_-coupled receptor calcitonin receptor–like receptor (CALCRL), resulting in cAMP formation and PKA-mediated eNOS phosphorylation in an AKT-independent manner (25). However, role of adrenomedullin and G_s_-mediated signaling in the endothelial response to disturbed flow is still unknown.

Here, we show that disturbed flow also induces activation of the adrenomedullin/CALCRL/G_s_ system, which results in antiinflammatory downstream signaling in vitro and in vivo. Inhibition of this pathway in mouse endothelial cells in vivo promotes progression of atherosclerosis.

## Results

### Disturbed flow–induced endothelial inflammatory signaling is enhanced in the absence of G_s_-mediated signaling.

**Figure 1:**
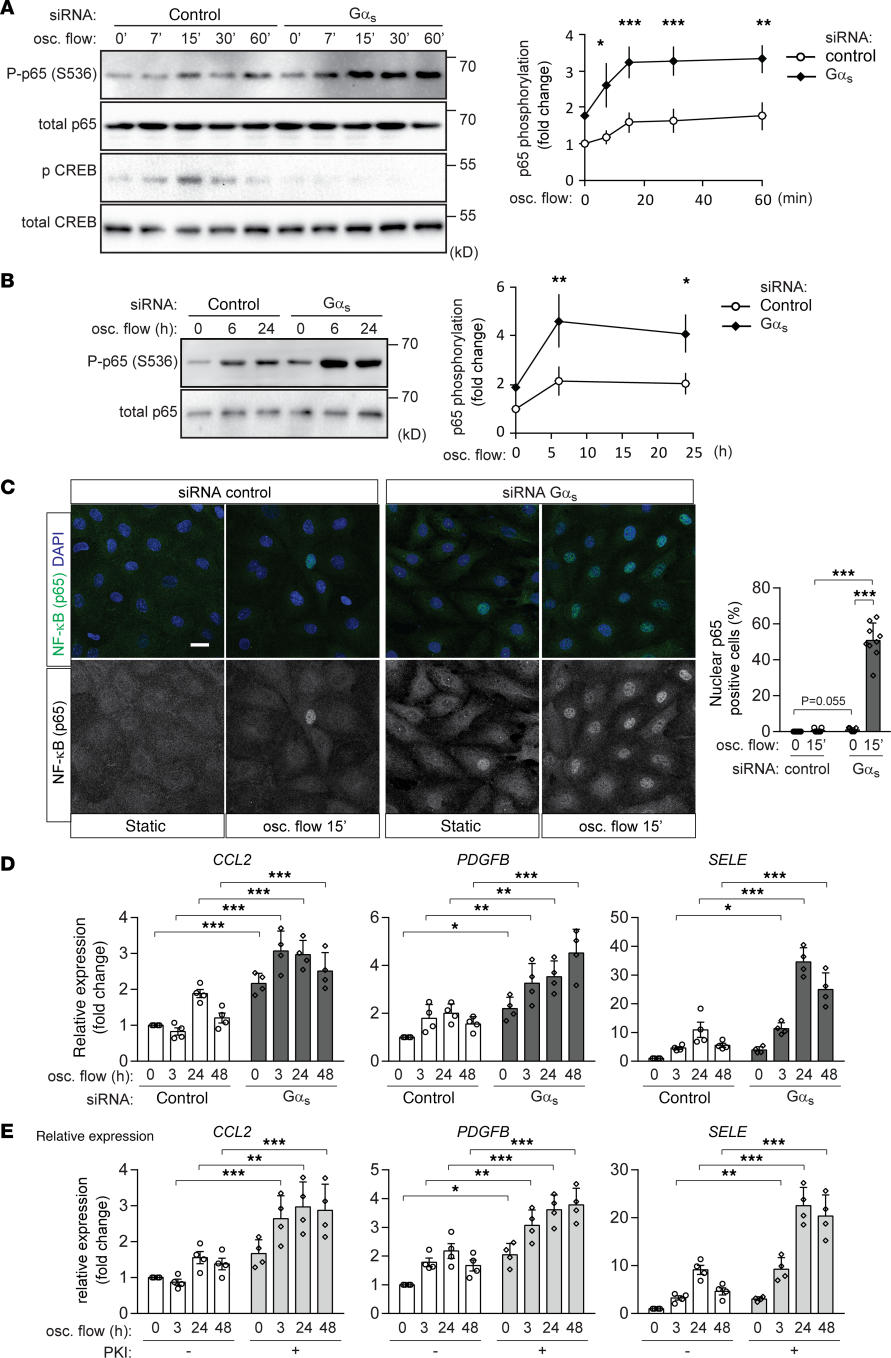
Knockdown of Gα_s_ increases endothelial inflammation induced by disturbed flow in BAECs. (**A**–**D**) Confluent BAECs were transfected with control siRNA or siRNA directed against Gα_s_ and were then exposed to oscillatory (osc.) flow for the indicated time period. p65 and/or CREB phosphorylation was analyzed by immunoblotting (**A** and **B**, *n* = 3 independent experiments), cellular p65 localization was determined by staining of cells with an anti-p65 antibody (**C**, *n* = 3 independent experiments, at least 3 view fields were analyzed per experiment), and inflammatory gene expression was analyzed by qPCR (**D**, *n* = 4 independent experiments). The diagrams show the densitometric evaluation of p65 phosphorylation (**A** and **B**) or of nuclear p65 staining (**C**). Scale bar: 20 μm. (**E**) Confluent BAECs were incubated without or with the PKA-inhibitor PKI (1 μM). Inflammatory gene expression after osc. flow induction was analyzed (*n* = 4 independent experiments). Data represent mean values ± SD; *P ≤ 0.05, **P ≤ 0.01, ***P ≤ 0.001 (2-way ANOVA and Bonferroni’s post hoc test [**A**, **B**, **D**, and **E**] and 2-tailed Student’s *t* test [**C**]).

**Figure 2:**
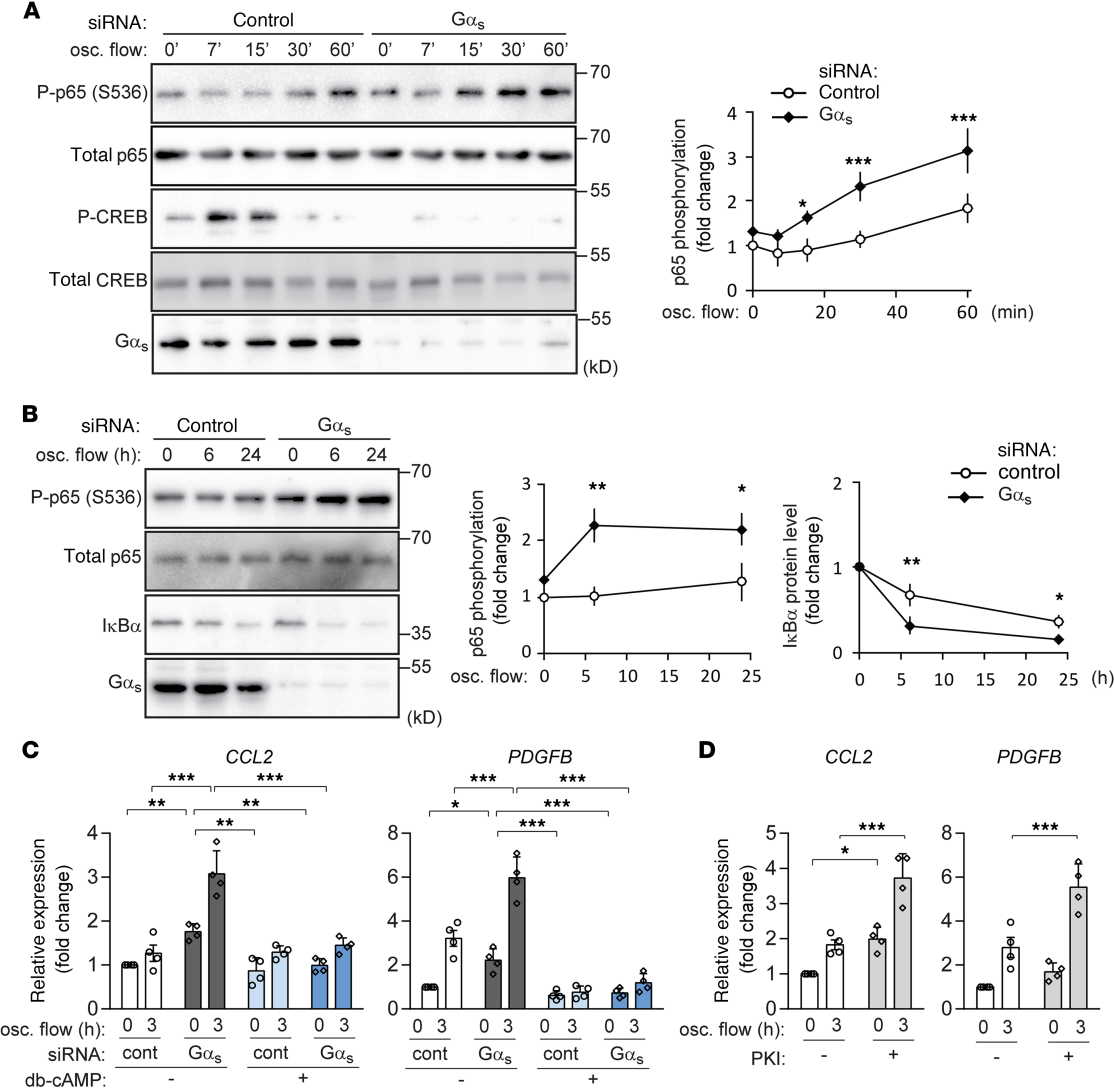
Endothelial inflammation induced by disturbed flow is suppressed by cAMP-PKA signaling in HUVECs. (**A** and **B**) Confluent HUVECs were transfected with control siRNA or siRNA directed against Gα_s_ and were then exposed to oscillatory (osc.) flow for the indicated time periods. NF-κB (p65) phosphorylation, as well as CREB phosphorylation (**A**) or levels of IκBα (**B**), were determined by immunoblotting. The diagrams show the densitometric evaluation of immunoblots (*n* = 3 independent experiments). (**C**) Control or Gα_s_ knockdown HUVECs were incubated without or with 50 μM db-cAMP for 30 minutes before induction of osc. flow (3 hours). Inflammatory gene expression was analyzed by qPCR (*n* = 4 independent experiments). (**D**) Confluent HUVECs were incubated without or with the PKA-inhibitor PKI (1 μM), followed by induction of osc. flow, and inflammatory gene expression (*n* = 4 independent experiments) was determined. Data represent mean values ± SD; *P ≤ 0.05, **P ≤ 0.01, ***P ≤ 0.001 (2-way ANOVA and Bonferroni’s post hoc test).

To test the role of G_s_-mediated signaling in the endothelial response to disturbed flow, we performed an siRNA-mediated knockdown of Gα_s_, the α subunit of the G_s_, and exposed cells to oscillatory flow. In control bovine aortic endothelial cells (BAECs), disturbed flow induced NF-κB activation as indicated by phosphorylation of p65 at serine 536 (Figure 1, A and B), which was further increased when flow chambers were coated with exogenous fibronectin (Supplemental Figure 1, A and B; supplemental material available online with this article; https://doi.org/10.1172/jci.insight.140485DS1). Disturbed flow also induced an early transient phosphorylation of cAMP response element binding protein (CREB), a target of cAMP-mediated signaling (Figure 1A). As expected, disturbed flow–induced CREB phosphorylation was blocked after knockdown of Gα_s_ (Figure 1A). Gα_s_ knockdown also resulted in increased levels of p65 phosphorylation (Figure 1, A and B), an effect also seen after coating of flow chambers with exogenous fibronectin (Supplemental Figure 1B). An increased endothelial NF-κB activation in the absence of G_s_-mediated signaling was also indicated by enhanced nuclear translocation of p65 after exposure to oscillatory flow (Figure 1C). Since activation of NF-κB has been shown to induce expression of inflammatory molecules in endothelial cells, we performed quantitative PCR (qPCR) to test whether knockdown of Gα_s_ also affects their expression. As shown in Figure 1D, loss of G_s_-mediated signaling in BAECs resulted in an increased expression of inflammatory genes such as CCL2, PDGFB, and E-selectin. Similar to p65 phosphorylation, inflammatory gene expression in control cells was increased after coating of flow chambers with fibronectin, while expression after Gα_s_ knockdown increased under both conditions (Supplemental Figure 1C). Flow chambers without additional fibronectin coating were used for further experiments. The effect of a Gα_s_ knockdown was mimicked by incubation of cells with the specific myristoylated PKA inhibitor peptide PKI (26, 27) (Figure 1E). A similar increase of flow-induced NF-κB activation, as well as IκBα degradation after knockdown of Gα_s_ was observed in HUVECs (Figure 2, A and B). qPCR using HUVECs also showed that both knockdown of Gα_s_, as well as inhibition of PKA, increased expression of CCL2 and PDGFB (Figure 2, C and D and Supplemental Figure 1D), and incubation of cells with dibutyryl-cAMP, a membrane-permeable stable derivative of cAMP, suppressed inflammatory gene expression induced by disturbed flow (Figure 2C). In contrast, the effects of TNF-α on NF-κB activation, IκBα degradation and CREB phosphorylation were not affected by loss of G_s_-mediated signaling in HUVECs (Supplemental Figure 2A), indicating that the proinflammatory effects of Gα_s_/cAMP signaling were specific for disturbed flow–induced inflammatory responses.

It has previously been shown that disturbed flow–induced inflammatory signaling including NF-κB activation involves activation of integrin α5 and FAK (17–24). We therefore tested whether knockdown of Gα_s_ affects disturbed flow–induced activation of integrin α5. However, disturbed flow–induced integrin α5 activation was not changed after loss of G_s_-mediated signaling in endothelial cells (Supplemental Figure 2B), indicating that cAMP/PKA-mediated signaling induced through G_s_ exerts its antiinflammatory effects downstream of integrin α5. The activities of other known upstream regulatory molecules of NF-κB such as Akt (28, 29) or GSK3b (30) also showed no difference in the absence of G_s_-mediated signaling (Supplemental Figure 2B), while IκBα degradation was facilitated after Gα_s_ knockdown in endothelial cells (Figure 2B). Since we have recently shown that G_s_-mediated signaling plays a critical role in mediating laminar flow–induced formation of NO (25), which can inhibit NF-κB activity via several mechanisms (31, 32), we further examined eNOS activation. Disturbed flow had a very small but significant stimulatory effect on eNOS phosphorylation and NO-formation, which was attenuated by loss of G_s_-mediated signaling (Supplemental Figure 3, A and B). However, inhibition of NO-formation by the NOS-inhibitor N(G)-Nitro-L-arginine methyl ester (L-NAME) did not affect disturbed flow–induced inflammatory signaling (Supplemental Figure 3, C and D).

### Endothelial G_s_-deficiency results in increased endothelial inflammation and progression of atherosclerosis in vivo.

**Figure 3:**
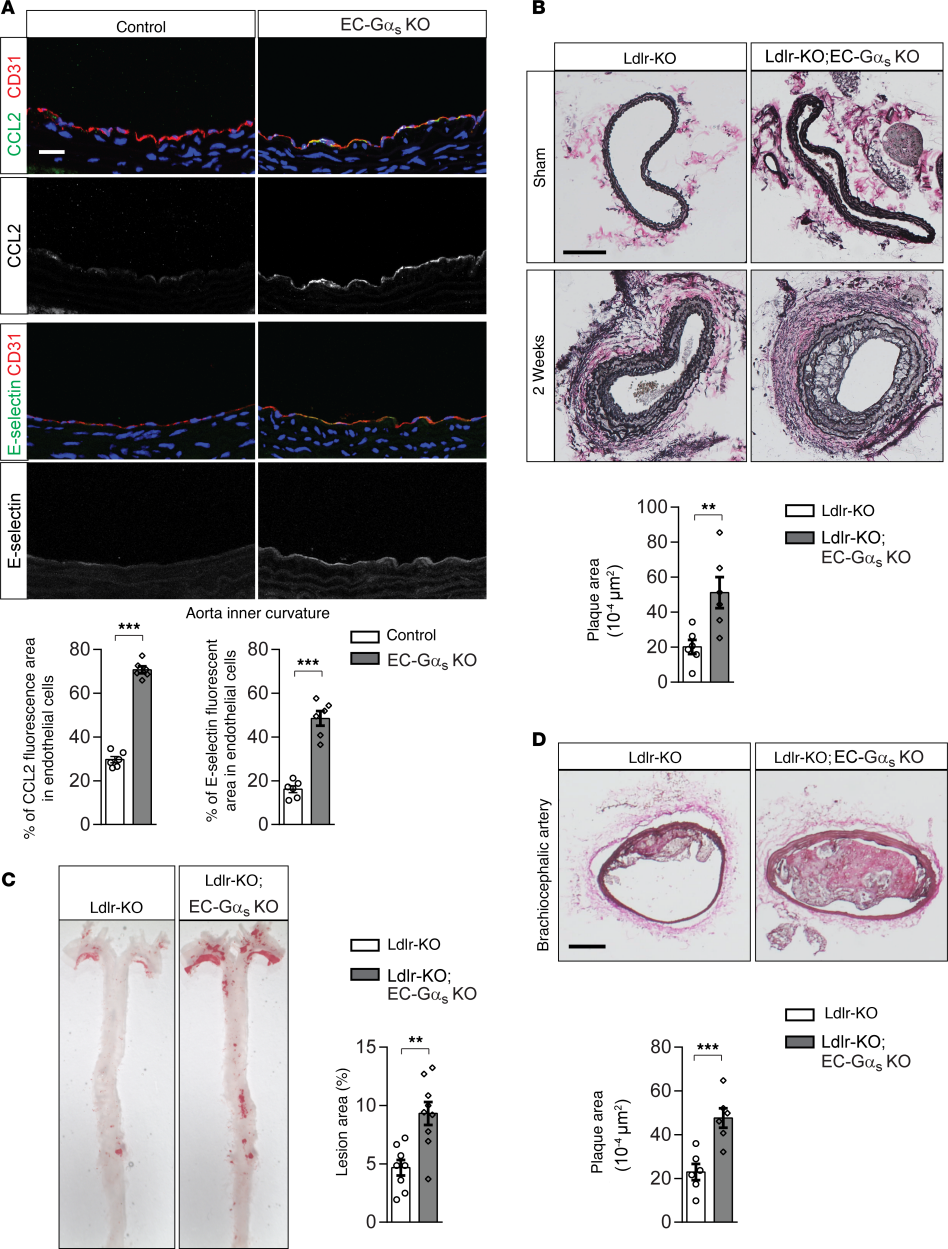
Induced loss of endothelial Gα_s_ results in increased endothelial inflammation and atherosclerotic plaque formation. (**A**) Cross-sections of the inner curvatures of aortic arches of control or EC-Gα_s_–KO mice stained with DAPI (blue) and antibodies against CCL2 or E-selectin (green) and CD31 (red). Bar diagrams show percentage of area stained by anti-CCL2 or anti–E-selectin antibodies of total endothelial cell area defined by anti-CD31 antibody staining (*n* = 6 animals per group; at least 3 sections were analyzed per animal). Scale bar: 25 μm. (**B**) Atherosclerosis-prone Ldlr-KO mice without (Ldlr-KO) or with endothelium-specific Gα_s_ deficiency (Ldlr-KO;EC-Gα_s_–KO) were sham operated or underwent partial carotid artery ligation. Two weeks after the ligation, serial sections were made through the entire carotid arteries and stained with elastic stain (*n* = 6 mice per group). Scale bar: 100 μm. (**C** and **D**) Ldlr-KO or Ldlr-KO;EC-Gα_s_–KO mice were fed a high-fat diet for 14 weeks. (**C**) En face view on whole aortae stained with Oil Red O. (**D**) Representative images of atherosclerotic plaques observed in brachiocephalic arteries. Scale bar: 50 μm. The bar diagrams show atherosclerotic lesion area as percentage of total aorta area (**C**) or total arterial area (**D**) (*n* = 8, Ldlr-KO; *n* = 9, Ldlr-KO;EC-Gα_s_–KO [**C**]; *n* = 6 per genotype [**D**]). Data represent mean ± SEM; **P ≤ 0.01, ***P ≤ 0.001 (2-tailed Student’s *t* test).

Next, we examined whether endothelial G_s_-mediated signaling is involved in disturbed flow–induced endothelial inflammation in vivo by using tamoxifen-inducible endothelium-specific Gα_s_-deficient mice (Tek-CreERT2;Gnas^fl/fl^, herein referred to as EC-Gα_s_–KO mice) (25, 33). At the inner curvature of the aortic arch, where endothelial cells are naturally exposed to disturbed, atherogenic flow, endothelial expression of CCL2 and E-selectin was increased in EC-Gα_s_–KO mice compared with control animals (Figure 3A). CCL2 expression also tended to be increased in endothelial cells of the outer aortic curvature in the absence of Gα_s_ (Supplemental Figure 4A). We then studied the function of endothelial G_s_-signaling in atherogenesis by performing partial carotid artery ligation, a model of endothelial dysfunction and atherosclerosis that is acutely induced by disturbed flow and reduced laminar flow (34). Two weeks after partial carotid artery ligation, the neointima size was significantly increased in LDL-receptor–deficient mice with endothelium-specific Gα_s_ deficiency compared with LDL-receptor–deficient control animals (Figure 3B). Similar results were obtained in a long-term model of atherosclerosis. After 14 weeks of feeding a high-fat diet, the progression of atherosclerosis was significantly increased in the aorta and the innominate artery of EC-Gα_s_–KO mice lacking the LDL-receptor compared with control LDL-receptor–deficient animals (Figure 3, C and D). Infiltration of CD68^+^ cells in the plaque lesion was significantly increased in LDL-receptor–deficient mice with endothelium-specific Gα_s_ deficiency (Supplemental Figure 4B).

### The adrenomedullin receptor CALCRL mediates disturbed flow–induced antiinflammatory effects.

**Figure 4:**
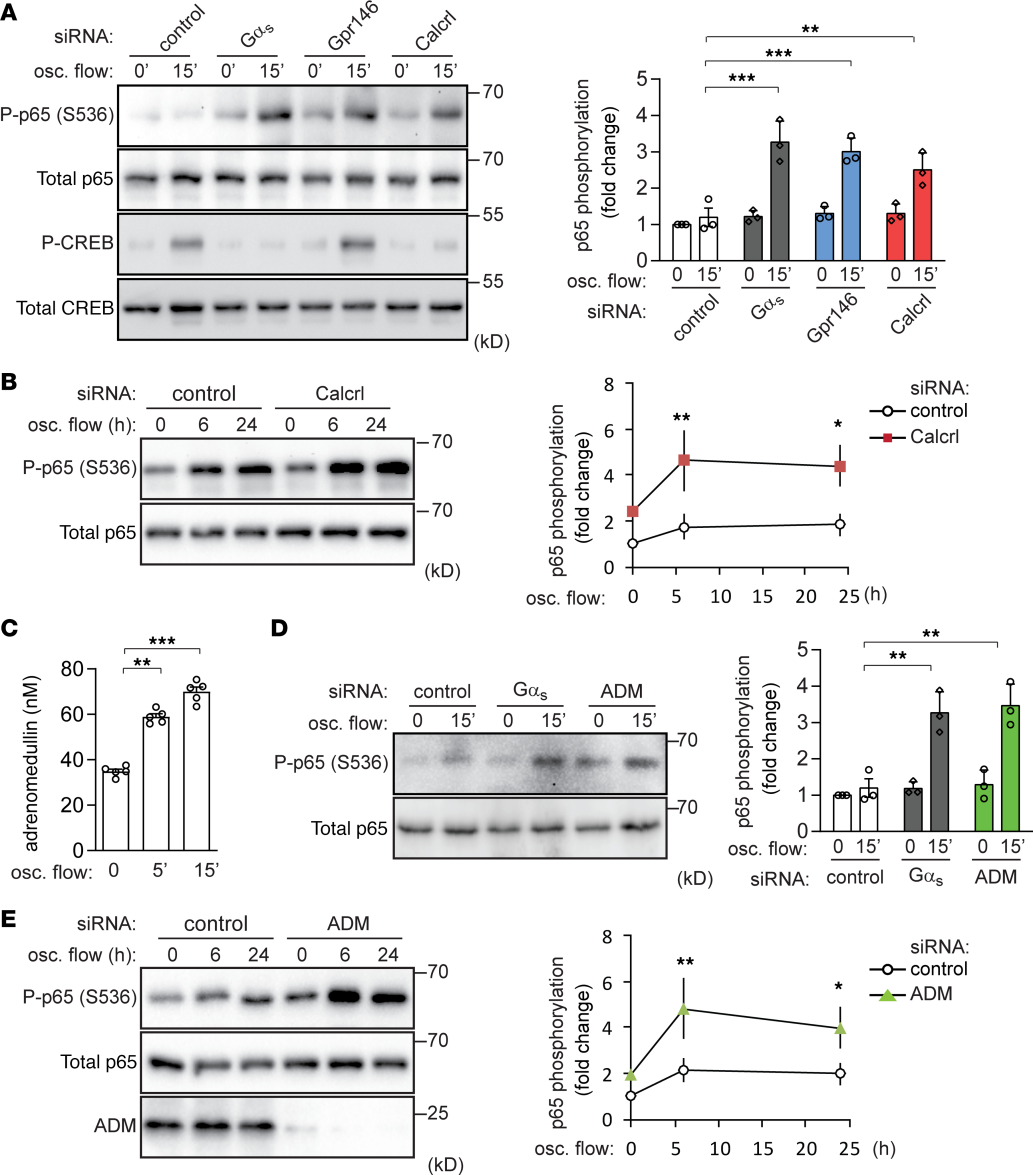
Adrenomedullin is released in response to oscillatory flow and activate antiinflammatory signaling though its receptor CALCRL. (**A** and **B**) Confluent BAECs were transfected with control siRNA (control) or siRNA directed against Gα_s_, GPR146, or CALCRL. p65 and CREB phosphorylation before or after oscillatory (osc.) flow induction (15 minutes) were determined. Bar diagrams show the statistical evaluation of immunoblots (*n* = 3 independent experiments). (**C**) Confluent BAECs were exposed to osc. flow for the indicated time periods. Thereafter, adrenomedullin concentration in the cell culture medium was determined (*n* = 3 independent experiments). (**D** and **E**) Control, Gα_s_-knockdown, or adrenomedullin-knockdown (ADM-knockdown) BAECs were exposed to osc. flow, and p65 phosphorylation was determined by immunoblotting. Bar diagrams show the statistical evaluation of immunoblots (*n* = 4 independent experiments). Data represent mean ± SD; *P ≤ 0.05, **P ≤ 0.01, ***P ≤ 0.001 (2-way ANOVA and Bonferroni’s post hoc test [**A**, **B**, **D**, and **E**] and 1-way ANOVA and Tukey’s post hoc test [**C**]).

Since role of G_s_ in disturbed flow–induced antiinflammatory effects in endothelial cells strongly suggests the involvement of a GPCR operating upstream of G_s_, we performed an siRNA-mediated knockdown of several G_s_-coupled receptors or orphan receptors that had previously been shown to be highly expressed in HUVECs, BAECs, and murine aortic ECs (25). In BAECs, knockdown of the CALCRL and of the orphan receptor Gpr146 resulted in an increased disturbed flow–induced p65 phosphorylation to a similar extent as knockdown of Gα_s_, while suppressed expression of other candidate receptors had no effect (Supplemental Table 1). An alternative siRNA against the RNA encoding these GPCRs showed a similar increase of NF-κB activation (Figure 4, A and B). However, knockdown of Gpr146 did not affect disturbed flow–induced CREB phosphorylation (Figure 4A), suggesting a G_s_-independent effect. We therefore focused on CALCRL as a potential receptor mediating disturbed flow–induced G_s_-dependent antiinflammatory effects in endothelial cells.

CALCRL functions together with receptor activity–modifying protein 2 (RAMP2) as a receptor for adrenomedullin (35), which has recently been shown to be released from endothelial cells in response to laminar flow in a manner depending on the mechanosensitive cation channel PIEZO1 (25). Similar to laminar flow, oscillatory flow was also able to induce adrenomedullin release from endothelial cells (Figure 4C), which was significantly reduced by PIEZO1 knockdown (Supplemental Figure 5A). We therefore tested the effect of adrenomedullin knockdown on the endothelial response to disturbed flow, and we found that loss of endothelial adrenomedullin also resulted in a similar increase of disturbed flow–induced NF-κB activation as endothelial Gα_s_ (Figure 4, D and E). In addition, knockdown of CALCRL or adrenomedullin, or blockade of the CALCRL by the competitive adrenomedullin antagonist adrenomedullin 22–52 (AM22–52), increased disturbed flow–induced expression of inflammatory genes in BAECs (Supplemental Figure 5, B–D). However, as shown before (24), suppression of PIEZO1 expression rather suppressed p65 phosphorylation (Supplemental Figure 5E), which is most likely due to its predominating role in mediating disturbed flow–induced proinflammatory signaling (24). Nevertheless, these data indicate that both adrenomedullin and its receptor CALCRL are required for disturbed flow–induced G_s_-mediated antiinflammatory effects.

### Endothelial adrenomedullin and CALCRL mediate antiinflammatory and antiatherogenic effects in vivo.

**Figure 5:**
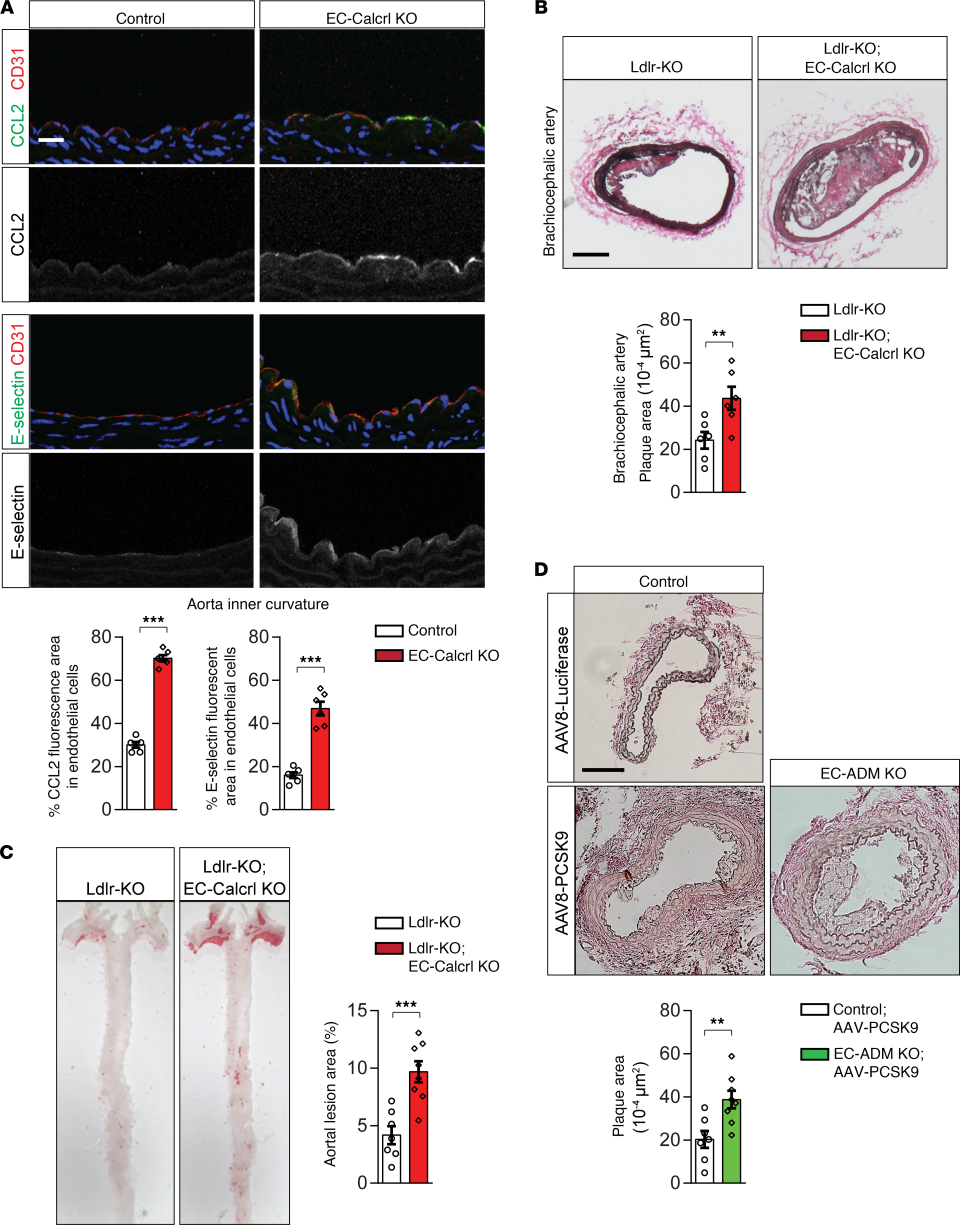
Loss of endothelial ADM and its receptor CALCRL results in increased endothelial inflammation and atherosclerotic plaque formation. (**A**) Cross-sections of the inner curvatures of aortic arches of control and EC-CALCRL–KO mice stained with DAPI (blue) and antibodies against CCL2 (green), E-selectin (green), or CD31 (red). Bar diagrams show percentage of area stained by anti-CCL2 or anti–E-selectin antibodies of total endothelial cell area stained by anti-CD31 antibody (*n* = 6 mice per group). Scale bar: 25 μm. (**B** and **C**) Atherosclerosis-prone Ldlr-KO mice without (Ldlr-KO) or with induced endothelium-specific deficiency of CALCRL (Ldlr-KO;EC-CALCRL–KO) were fed a high-fat diet for 14 weeks. (**B**) Representative images of Oil Red O–stained brachiocephalic artery. Plaque area was quantified as percentage of total aortic area (*n* = 6 mice per group). (**C**) En face view on atherosclerotic lesions of aortae stained with Oil Red O (*n* = 7, Ldlr-KO; *n* = 8, Ldlr-KO;EC-CALCRL–KO). Scale bar: 200 μm. (**D**) Control or EC-Adm–KO mice were injected with AAV-PCSK9 or with AAV-Luc i.v. 1 week before partial carotid artery ligation. Two weeks after the ligation, serial sections were made through the entire carotid arteries and stained with elastic stain (*n* = 6, control; *n* = 8, EC-ADM KO). Scale bar: 100 μm. Data represent mean ± SEM; **P ≤ 0.01, ***P ≤ 0.001 (2-tailed Student’s *t* test).

Finally, we examined whether endothelial adrenomedullin and its receptor are involved in disturbed flow–induced endothelial inflammation in vivo. In induced endothelium-specific CALCRL-deficient mice (Tek-CreERT2;Calcrl^fl/fl^, herein referred to as EC-CALCRL–KO mice) (25), we found increased expression of inflammatory genes such as CCL2 and E-selectin in endothelial cells of the inner curvature of the aorta (Figure 5A). Furthermore, when crossed to LDL-receptor–deficient mice, EC-CALCRL–KO mice recapitulated the phenotype of EC-Gα_s_–KO mice after feeding a high-fat diet, showing an increased progression of atherosclerosis in the aorta, as well as in the innominate artery (Figure 5, B and C). We also analyzed atherosclerosis progression in endothelium-specific adrenomedullin–deficient mice (Tek-CreERT2;Adm^fl/fl^, herein referred to as EC-Adm–KO mice) (25) treated with adeno-associated virus (AAV) transducing proprotein convertase subtilisin-kexin type 9 (PCSK-9) (AAV-PCSK9) (36) to induce hypercholesterolemia (Supplemental Figure 6, A and B). Similar to EC-Gα_s_–KO mice lacking an LDL receptor, the neointima size in hypercholesterolemic EC-Adm–KO mice was significantly increased compared with control animals 2 weeks after partial carotid ligation (Figure 5D).

## Discussion

Laminar and disturbed flow induce profoundly different effects in endothelial cells, resulting in an anti- or proatherogenic phenotype, respectively (4–9, 12, 37). While disturbed flow promotes inflammatory signaling pathways — including NF-κB activation, which results in the expression of various leukocyte adhesion molecules such as E-selectin and VCAM-1, as well as chemokines such as CCL2 (13–15) — laminar flow rather induces activation of eNOS (38–40). Both signaling pathways involve, in part, the same components, including activation of the purinergic P2Y_2_ receptor and G_q_/G_11_-mediated signaling (24, 41), as well as a mechanosignaling complex consisting of PECAM-1, VE-cadherin, and VEGFR2 (16, 41). It has recently been shown that laminar flow activates the G_s_-mediated signaling pathway and subsequent eNOS phosphorylation and activation by promoting adrenomedullin release from endothelial cells and activation of its receptor CALCRL (25). In the present study, we show that disturbed flow also promotes the release of adrenomedullin from endothelial cells, as well as the subsequent activation of the G_s_-coupled adrenomedullin receptor on endothelial cells, resulting in the inhibition of proinflammatory signaling, including NF-κB activation. Thus, both laminar and disturbed flow induce atheroprotective downstream signaling events through CALCRL/G_s_-mediated signaling. In contrast, P2Y_2_/G_q_/G_11_-mediated signaling induced by laminar flow promotes atheroprotective downstream signaling, such as eNOS activation, but activation of this pathway by disturbed flow leads to proinflammatory signaling, including NF-κB activation (24, 41). This indicates that modulating the endothelial adrenomedullin/CALCRL/G_s_ system is a more promising approach to inhibit progression of atherosclerosis.

In our previous and present studies, we showed that PIEZO1 is involved not only in oscillatory flow–induced proinflammatory signaling by mediating ATP and G_q_/G_11_-dependent NF-κB activation (24), but also in antiinflammatory signaling by mediating adrenomedullin and G_s_-dependent cAMP generation. Oscillatory flow–induced NF-κB activation is blocked after PIEZO1 knockdown (24), since cAMP inhibits disturbed flow–induced NF-κB activation, and its effect depends on PIEZO1-mediated induction of inflammatory signaling.

Several mechanisms may underlie the inhibition of inflammatory signaling through NF-κB by adrenomedullin/CALCRL/G_s_-mediated cAMP formation (42). Increases in intracellular cAMP levels in response to laminar flow have been shown to result in PKA-dependent phosphorylation and activation of eNOS, whose product, NO, can inhibit NF-κB activity via several mechanisms (31, 32, 43). However, our data indicate that eNOS phosphorylation and endothelial NO formation in response to disturbed flow is rather small compared with the effect of laminar flow, making it unlikely that NO is a critical mediator in the antiinflammatory effect of G_s_/cAMP signaling. In addition, blockade of NO formation by L-NAME did not promote expression of inflammatory genes under basal conditions or after disturbed flow. In contrast, inhibition of PKA promoted disturbed flow–induced p65 phosphorylation and increased basal as well as disturbed flow–induced expression of inflammatory genes. It is therefore more likely that cAMP produced through activation of the CALCRL/G_s_ signaling pathway in response to disturbed flow inhibits NF-κB signaling via PKA, affecting downstream integrin signaling events, on the same level as or upstream of IκB protein degradation. Moreover, elevation of cAMP has also been shown to inhibited NF-κB–mediated transcriptional regulation in endothelial cells (44).

Several GPCRs, including the sphingosine-1-phosphate receptor S1P_1_, GPR68, and bradykinin B2 are known to respond to flow in a ligand-independent manner (45–47). Since the competitive adrenomedullin receptor antagonist AM22–52 mimics the effect of a knockdown of CALCRL or of Gα_s_ on disturbed flow–induced inflammatory signaling, it is more likely that disturbed flow–induced CALCRL activation requires the presence of the adrenomedullin ligand. This is consistent with previous findings and our observation that different flow patterns, including disturbed flow, are able to induce the expression and/or release of adrenomedullin (25, 48–50). A critical role of adrenomedullin in disturbed flow–induced inhibition of inflammatory signaling through activation of CALCRL is also supported by our observation that suppression of endothelial adrenomedullin expression in vitro and in vivo promotes disturbed flow–induced inflammatory signaling and atherosclerosis progression.

The fact that mice lacking adrenomedullin, CALCRL, or Gα_s_ in endothelial cells show an increased progression of atherosclerosis indicates that this signaling pathway is constantly activated in areas of disturbed flow. It has previously been shown that systemic administration of adrenomedullin or transgenic overexpression of adrenomedullin reduces the progression of atherosclerosis in an ApoE-deficient mouse line (51, 52). Our data provide a mechanism for those observed effect and further support the concept that activation of the adrenomedullin/CALCRL/G_s_-mediated signaling system has beneficial atheroprotective effects.

## Methods

### Reagents.

Adrenomedullin, L-NAME, and fibronectin were purchased from Sigma-Aldrich (catalogs A-2327, N-5751, F-4759). Myristoylated PKA inhibitor 14–22 (PKI) and dibutyryl-cAMP were purchased from Merck Chemicals GmbH (catalogs 476485, 28745). Human AM22–52 was purchased from Bachem (catalog H-4144). Human recombinant TNF-α was purchased from PeproTech (catalog 300-01A).

### AAV vectors preparation.

AAV serotype 8 vectors expressing the murine D377Y-PCSK9 cDNA (AAV-PCSK9) or luciferase as control (AAV-Luc) were produced using the 2-plasmid method by cotransfecting AAV/D377Y-mPCSK9 (gift from Jacob Bentzon; Addgene plasmid no. 58376) (36) or pSSV9-CMV-Luc together with the helper plasmid pDP8 (53) in HEK293T cells using polyethylenimine (Sigma-Aldrich), and they were purified using iodixanol step gradients and titrated as previously described (54).

### Primary cells.

HUVECs and BAECs were purchased from Lonza. HUVECs were cultured with EGM-2 (Lonza), and BAECs were cultured with endothelial growth medium (EGM-2-MV, Lonza). Confluent cells at passage ≤ 6 were used in all experiments.

### siRNA-mediated knockdown.

Cells at 70% confluence were transfected with siRNAs by using Opti-MEM (Thermo Fisher Scientific) and Lipofectamine RNAiMAX (Invitrogen) as described previously (41). SiRNAs used for the screen were described previously (25). The targeted sequences of other siRNAs were as follows: human *Gnas* 5′-CUGAUUGACUGUGCCCAGU-3′; bovine *Gnas* 5′-CTGATTGACTGCGCCCAGT-3′; bovine *Calcrl* 5′-GAATCCAATCTGTACATGA-3′, 5′-GACTATAATTGGACATGTA-3′, and 5′-GATCAGTTCTGATACGCAA-3′; bovine *Adm1* 5′-GCATCCGAGTCAAGCGCTA-3′, 5′-CCGCCAGAGTTTGAACAAC-3′, and 5′-GGACATGCACGGTGCAGAA-3′; and bovine *Piezo1* 5-GTGTTTGGTCTCAAGGACT-3, 5-GTGTCTACTTCCTGCTCTT-3. Multiple sequences indicate pooled siRNAs (mix of different siRNAs targeting a single gene).

### Flow experiments.

For biochemical experiments, cells were seeded in μ-slide I^0.4^ Luer chambers (Ibidi, 80176). Flow chambers were coated with fibronectin (1 μg/cm^2^) if indicated. Oscillatory flow (5 dynes/cm^2^ with a frequency of 1 Hz) was applied on confluent monolayers using a parallel-plate flow chamber (μ-slide I^0.4^ Luer, Ibidi). During the experiments, flow units were maintained at 37°C in a humidified atmosphere with 5% CO_2_ in a tissue culture incubator. For experiments to determine cAMP, nitrate/nitrite, and adrenomedullin levels, the BioTech-Flow System cone-plate viscometer (MOS Technologies) was used. The cone of this system has an angle of 2.5° and rotates on top of a 33 cm^2^ cell culture dish containing 3 mL of medium. Shear stress was calculated with the following formula (assuming a Reynolds number of << 1): τ = η × 2π × n/0.044, where τ represents shear stress, η represents viscosity, and n represents rotational speed. Oscillatory flow was set at 4 dynes/cm^2^ (at a rotation speed of 28 rpm, 40 amplitude, and 1 HZ) as described previously (24).

### Western blot analysis.

Total protein was extracted from cells using RIPA buffer supplemented with protease inhibitors (10 mg/mL of leupeptin, pepstatin A, 4-[2-aminoethyl]benzensulfonylfluorid and aprotinin), and phosphatase inhibitors (PhosSTOP, Roche) as described (25). Total cell lysates were subjected to SDS-PAGE electrophoresis and transferred to nitrocellulose membranes or PVDF membrane. After blocking (5% BSA or 0.3% skim milk in TBST at room temperature for 30 minutes), the membranes were incubated with gentle agitation overnight at 4°C with the primary antibodies: anti–NF-κB P65 (Cell Signaling Technology, 4764), anti–phospho-P65 (Cell Signaling Technology, 3033), anti-CREB (Cell Signaling Technology, 9197), anti–phospho-CREB (Thermo Fisher Scientific, MA5-11192), anti-AKT (Cell Signaling Technology, 9272), anti–phospho-AKT (Cell Signaling Technology, 4060), anti-GSK3β (Cell Signaling Technology, 9315) anti–phospho-GSK3β (Cell Signaling Technology, 5558), anti-SAPK/JNK (Cell Signaling Technology, 9252), anti–phospho-SAPK/JNK (Cell Signaling Technology, 9251), anti-IκBα (Cell Signaling Technology, 4812), anti–integrin α5 (Abcam, ab150361), anti-eNOS (BD Biosciences, 610296), anti–phospho-eNOS (human S1177, Cell Signaling Technology, 9571), anti–phospho-eNOS (human S633, BD Biosciences, 07-562), anti–low-density lipoprotein receptor (anti-LDLR; Thermo Fisher Scientific, MA532075). The membranes were then washed 3 times for 7 minutes each with TBST and incubated with HRP-conjugated secondary antibodies (Cell Signaling Technology, dilution 1:3000) followed by chemiluminescence detection using ECL substrate (Pierce) according to the manufacturer’s protocol. Band intensities from immunoblotting were quantified by densitometry using ImageJ (NIH) software (55).

### qPCR analysis.

Total RNA was isolated from endothelial cell monolayers using the Quick-RNA Micro prep kit (Zymo) according to the manufacturer’s protocol. Quality control of samples was carried out using a Nanodrop ND-100 Spectrophotometer. Complementary DNA synthesis was performed using the ProtoScript II Reverse Transcription kit (New England BioLabs, M0368S). qPCR was performed using primers designed with the online tool provided by Roche and the Light-Cycler 480 Probe Master System (Roche). Each reaction was run in triplicate, and relative gene expression levels were normalized to GAPDH. Relative expression was calculated using the ΔΔCt method. Primer sequences used were as follows: human *Gapdh* forward 5′-AGCCACATCGCTCAGACAC-3′, reverse 5′-GCCCAATACGACCAAATCC-3′; human *Ccl2(Mcp1)* forward 5′-AGTCTCTGCCGCCCTTCT-3′, reverse 5′-GTGACTGGGGCATTGATTG-3′; human *Pdgfb* forward 5′-CTGGCATGCAAGTGTGAGAC-3′, reverse 5′-CGAATGGTCACCCGAGTTT-3′; bovine *Gapdh* forward 5′-TCACCAGGGCTGCTTTTAAT-3′, reverse 5′- GAAGGTCAATGAAGGGGTCA-3′; bovine *Ccl2(Mcp1)* forward 5′-CGTTTGTTTTCATGGAGATTTG-3′, reverse 5′-TCCCAGGGATAGAACTGAGATT-3′; bovine *Pdgfb* forward 5′-GACTGATGGGGTCGCTGT-3′, reverse 5′-AGGGTTTCAGGCCAGTCC-3′; bovine *Sele* forward 5′-TGACCAAAGAAGCCACACAAACT-3′, reverse 5′-CACAGTCCTCATCACTTTGCTT-3′.

### Determination of p65 nuclear translocation.

BAECs were transfected with siRNAs in μ-slide I^0.4^ Luer slides (Ibidi). Twenty-four hours after the second siRNA transfection, cells were exposed to oscillatory flow for 15 minutes as described above and fixed in flow chambers for 10 minutes in 4% PFA. After permeabilization and blocking (0.3% Triton X-100 and 1% BSA in 1× PBS at room temperature for 1 hour), cells were incubated with primary antibody directed against NF-κB p65 (Cell Signaling Technology, 4764) overnight at 4°C (dilution 1:100). After gentle washing with PBS (3 times), cells were incubated with corresponding Alexa Fluor 488–conjugated secondary antibody (1:500; Invitrogen) together with DAPI (1 ng/mL; Invitrogen) for detection of nuclei for 2 hours at room temperature in the dark. Stained monolayers were analyzed using a confocal microscope (SP5 Leica).

### Determination of ADM level.

Supernatants were collected from endothelial cells grown in BioTech-Flow chambers before and after exposure to flow. Samples were transferred to precooled tubes, and cellular debris was removed by centrifugation (20,000*g*) for 5 minutes at 4°C. Adrenomedullin concentration was determined with the bovine adrenomedullin ELISA Kit (BIOZOL Diagnostica Vertrieb GmbH, LS-F6083-1) following the manufacturer’s instructions.

### Determination of nitrate and nitrite (NOx) levels.

To determine flow-induced NOx release from endothelial cells, cells were exposed to fluid shear stress using the BioTech-Flow System, and supernatants were collected at different time points. Samples were transferred to precooled tubes, and cellular debris was removed by centrifugation (20,000*g*) for 5 minutes at 4°C. NOx levels were determined using a nitrate/nitrite fluorometric assay kit (Cayman, 780051) according to the manufacturer’s instructions.

### Determination of intracellular cAMP levels.

Endothelial cells were plated on culture glass of the BioTech-Flow viscometer chambers. Following flow application, cells were lysed in 0.1M HCl supplemented with 0.1% Triton X-100 and incubated for 10 minutes in room temperature. Lysates were collected and centrifuged (600*g* for 10 minutes at 4°C) to pellet cellular debris. Intracellular cAMP values were measured using Direct cAMP ELISA kit (Enzo Life Sciences Inc., ADI-900-066) following the manufacturer′s instructions.

### Experimental mice.

All mice were backcrossed onto a C57BL/6N background (Charles River), and experiments were performed with littermates as controls. Male animals (8–12 weeks old) were used unless stated otherwise. Mice were housed under a 12-hour light/12-hour dark cycle, with free access to food and water and under specific pathogen–free conditions unless stated otherwise. The generation of inducible endothelium-specific Gα_s_–deficient mice (Tie2-CreERT2;Gnas^fl/fl^), endothelium-specific CALCRL–, and adrenomedullin-deficient mice (Tie2-CreERT2;Calcrl^fl/fl^ and Tie2-CreERT2;Adm^fl/fl^, respectively) was described previously (25). Cre-mediated recombination was induced by i.p. injection of tamoxifen (MilliporeSigma, T5648) dissolved in Miglyol 812 (1 mg per mouse per day) on 5 consecutive days.

For atherosclerosis studies, endothelium-specific Gα_s_– or CALCRL- deficient mice were crossed with the low-density lipoprotein receptor KO (Ldlr-KO) and were fed a high-fat diet for 14 weeks to induce development of atherosclerotic lesions. Diet contained 21% butterfat and 1.5% cholesterol (Ssniff, TD88137). For atherosclerosis studies in endothelium-specific adrenomedullin–deficient mice, mice were injected with 1 × 10^11^ vector genome copies (VG) of AAV-PCSK9 or AAV-Luc via the tail vein 3 days after the last tamoxifen injection.

### Partial carotid artery ligation.

Partial carotid artery ligation was performed as described before (34). In brief, 10 days after the last tamoxifen injection, mice were anesthetized by i.p. injection of ketamine (120 mg/kg, Pfizer) and xylazine (16 mg/kg, Bayer) and placed on a heated surgical pad. After hair removal, a midline cervical incision was made, and the left internal and external carotid arteries were exposed and partially ligated with 6.0 silk sutures (Serag-Wiessner), leaving the superior thyroid artery intact. Skin was sutured with absorbable 6.0 silk suture (CatGut), and animals were monitored until recovery in a chamber on a heating pad after the surgery. Animals were fed a high-fat diet for 2 weeks (Ssniff, TD88137), at which time their carotid arteries were harvested. To determine atherosclerotic lesions, left (partially ligated) and right (sham) carotid arteries were removed and fixed in 4% PFA overnight. Fixed vessels were embedded in paraffin. Serial sections (5 μm) were made through the entire carotid arteries, and a defined segment (1000–1500 μm from the ligature, unless otherwise stated) were stained with accustain elastic stain (MilliporeSigma) according to manufacturer’s instructions. Plaque area was calculated by subtracting the lumen area from the area circumscribed by the internal elastic lamina.

### Determination of serum cholesterol level.

Mouse blood samples were collected without using an anticoagulant and were then allowed to clot for more than 30 minutes at room temperature. After centrifugation (2000*g* for 15 minutes at 4°C), the serum was removed, and cholesterol concentration was determined using a Cholesterol Fluorometric Assay kit (Cayman, 10007640) following the manufacturer’s instructions.

### Morphological analysis of atherosclerotic plaques.

For en face Oil Red O staining of atherosclerotic plaques, aortae were fixed in 4% PFA overnight at 4°C after perfusion (4% PFA, 20 mM EDTA [Carl Roth, 8043], 5% sucrose in 15 mL PBS). Thereafter, connective tissue and the adventitia were removed, and vessels were cut, opened, and pinned en face onto a glass plate coated with silicon. After rinsing with distilled water for 10 minutes and subsequently with 60% isopropanol, vessels were stained en face with Oil Red O for 30 minutes with gentle shaking; they were rinsed again in 60% isopropanol and then in tap water for 10 minutes. Samples were mounted on the coverslips with the endothelial surface facing upward with glycerol gelatin aqueous mounting media (MilliporeSigma). The innominate arteries were dissected and embedded in optimal cutting temperature medium (Tissue-Tek). Frozen innominate arteries were mounted in a cryotome, and a defined segment 500–1000 μm distal from the origin of the innominate artery was sectioned (10 μm). Sections were then stained with accustain elastic stain kit (MilliporeSigma) according to the manufacturer’s protocol. Frozen aortic outflow tracts were sectioned (10 μm) and stained with Oil Red O for 30 minutes and mounted with glycerol gelatin. Photoshop CS5 extended software (Adobe) was used to measure atherosclerotic plaque sizes and luminal cross-sectional areas.

### Histology and immunostaining.

Mice were sacrificed by CO_2_ inhalation, and the chest cavity was opened for perfusion with 4°C PBS and 4% PFA in PBS. To analyze the endothelial layer of the inner and outer aortic curvature, aortic arches were cryosectioned (10 μm) and fixed with ice-cold acetone for 10 minutes. The innominate arteries were prepared as described above. Optimal cutting temperature tissue-freezing medium (Tissue-Tek) was removed by washing with PBS 3 times for 5 minutes each, and sections were immunostained with antibodies with antibodies against CD31 (BD Biosciences, 550274; Abcam, ab24590), CCL2 (LS-c169178, LSBio), E-selectin (MilliporeSigma, S9555), or CD68 (Bio-Rad, MCA1957) overnight at 4°C. After washing 3 times with PBS, bound primary antibodies were detected using Alexa Fluor 488– or Alexa Fluor 594–conjugated secondary antibodies (1:500; Invitrogen). DAPI (1 ng/mL; Invitrogen) was used to label cell nuclei. Sections were viewed with a confocal microscope (Leica, SP5). For quantification, the endothelial cell area was defined by CD31 staining using ImageJ software, and the fluorescence signal indicating Ccl2 and selectin was then calculated as percentage of total endothelial cell area.

### Statistics.

Statistical data analysis is included in every figure and described in detail on the respective figure legends. The exact *P* values and number of cells analyzed per condition (*n*) are stated in the figures and figure legends. All experiments were repeated at least 3 times (independent replicates); *n* represents a pool across these experimental replicates. Trial experiments or experiments done previously were used to determine sample size with adequate statistical power. Samples were excluded in cases where RNA/cDNA quality or tissue quality after processing was poor (below commonly accepted standards). All statistical analyses were performed using Prism 6 software (GraphPad). A level of *P* < 0.05 was considered significant and reported to the graphs. Comparisons between 2 groups were performed using unpaired 2-tailed Student’s *t* test, and multiple group comparisons at different time points were performed by 1-way ANOVA followed by Tukey’s post hoc test or 2-way ANOVA followed by Bonferroni’s post hoc test.

### Study approval.

All procedures involving animal care and use in this study were approved by the local animal ethics committees of the Regierungspräsidia Karlsruhe and Darmstadt.

## Author contributions

AN performed most in vitro and in vivo experiments, evaluated data, and wrote the manuscript. JAJ and GL performed the partial carotid artery ligation. KAR performed injection of the AAV vectors. AI helped with the siRNA screen and generated EC-CALCRL–KO mice. ST helped with the analysis of the role of Gpr146 in endothelial cells. LSW and MC provided the floxed Gα_s_ allele and discussed data. OJM provided AAV-PCSK9 and AAV-Luc and discussed data. SO supervised the study, discussed data, and wrote the manuscript.

## Supplementary Material

Supplemental data

## Version 1 12/03/2020

Electronic publication
