# Supplementary material for: Disturbed flow–induced Gs-mediated signaling protects against endothelial inflammation and atherosclerosis
Source: JCI Insight. 2020 Dec 3;5(23):e140485. doi: 10.1172/jci.insight.140485 (PMC7714404; doi:10.1172/jci.insight.140485)
Supplement: Supplemental data [file jciinsight-5-140485-s147.pdf]

Supplemental material

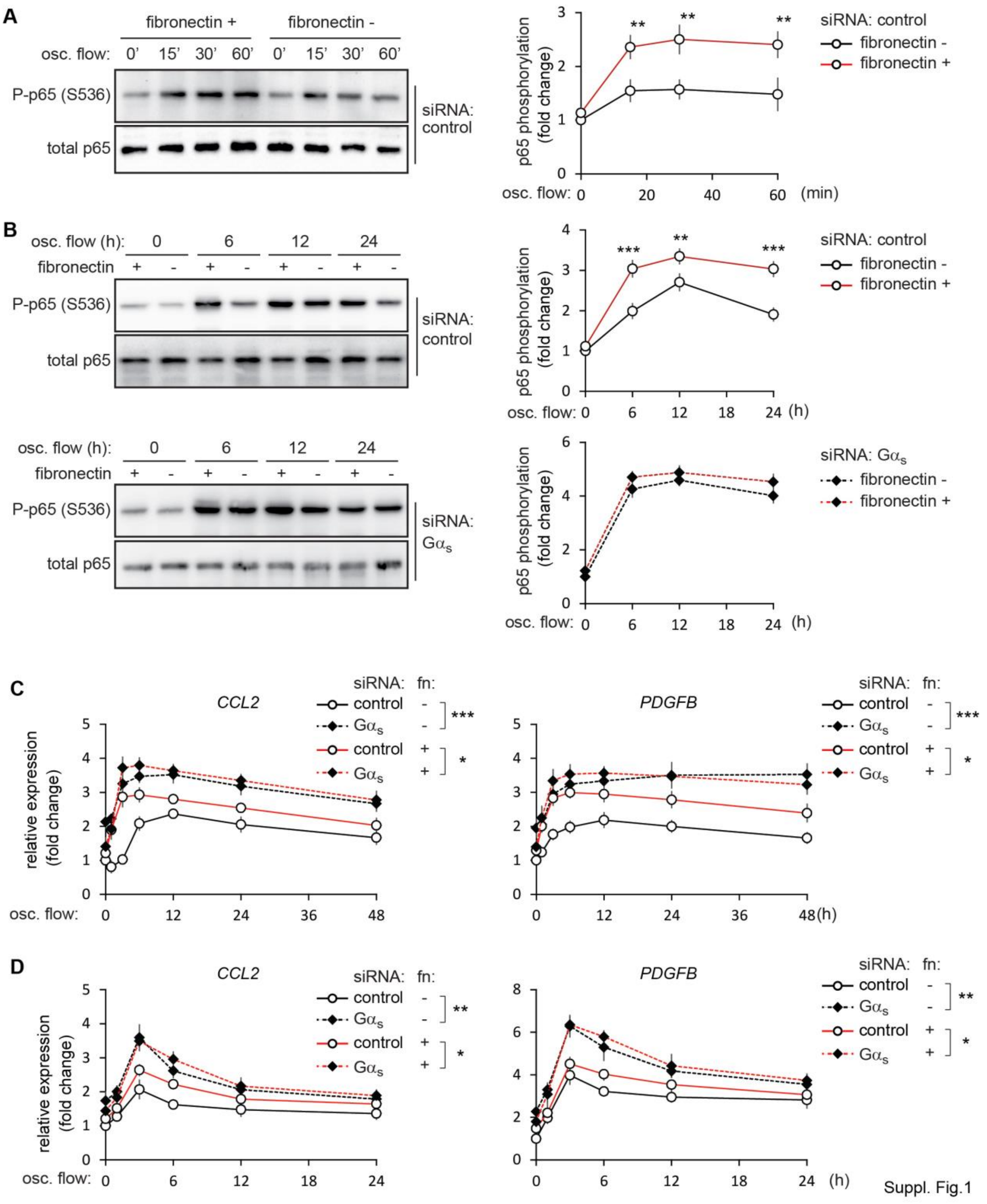

Suppl. Figure 1. Effect of the fibronectin coating of flow chambers on disturbed flow-induced endothelial inflammatory signaling. BAECs (A-C) or HUVECs (D) were seeded in flow chambers with or without fibronectin (fn) coating. After transfection of control siRNA (control) or siRNA directed against Gαs, oscillatory flow (5 dynes/cm^2

with a frequency of 1 Hz) was applied for the indicated time periods. **(A and B)** Levels of phosphorylated p65 was determined by immunoblotting. The diagrams show the densitometric evaluation of immunoblots (n=3 independent experiments). **(C and D)** Expression of inflammatory genes in BAEC (C) and in HUVECs (D) was analyzed by qRT-PCR (n=3 independent experiments). (Data represent mean values ± SD; * $P \leq 0.05$, ** $P \leq 0.01$, *** $P \leq 0.001$ (two-way ANOVA and Bonferroni's post hoc test)).

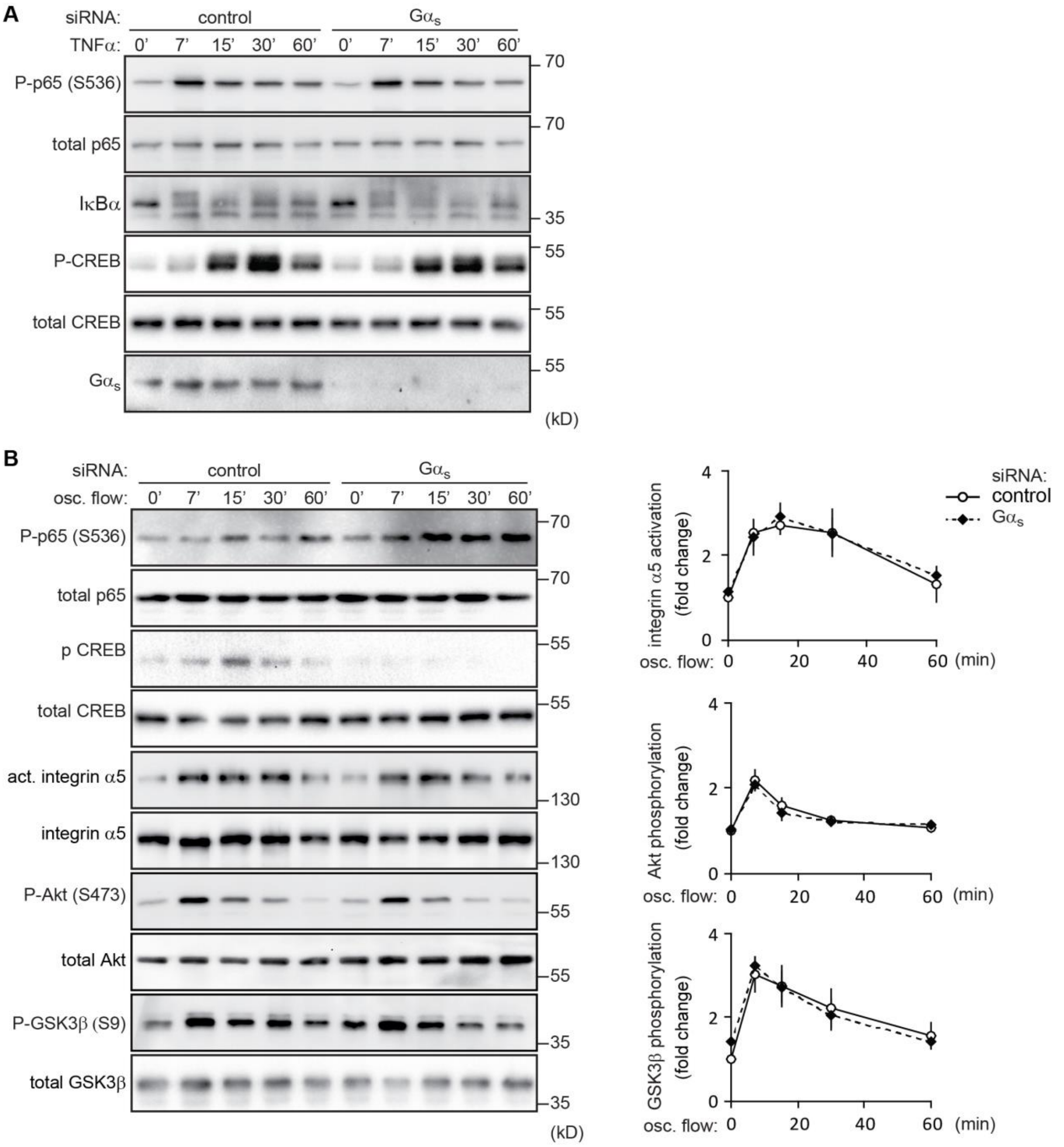

Suppl. Fig.2

Suppl. Figure 2. Effect of endothelial Gαs deletion on disturbed flow or TNFα induced signaling. (A) Control or Gαs knock-down HUVECs were incubated with TNFα (10 ng/ml) for the indicated time periods. Phosphorylation of p65 and CREB, as well as levels of IκBα and Gαs were determined by immunoblotting. **(B)** Control or Gαs knock-down BAECs were exposed to oscillatory (osc.) flow for the indicated time periods. Levels of phosphorylated p65, activated (act.) integrin α5 (antibody SNAKA51), phosphorylated AKT and GSK3β were determined by immunoblotting. The

diagrams show the densitometric evaluation of integrin α5 activation, AKT phosphorylation and GSK3β phosphorylation normalized to total integrin α5, AKT and GSK3β respectively (n=3 independent experiments). (Data represent mean values ± SD; *P ≤ 0.05, **P ≤ 0.01, ***P ≤ 0.001 (two-way ANOVA and Bonferroni's post hoc test)).

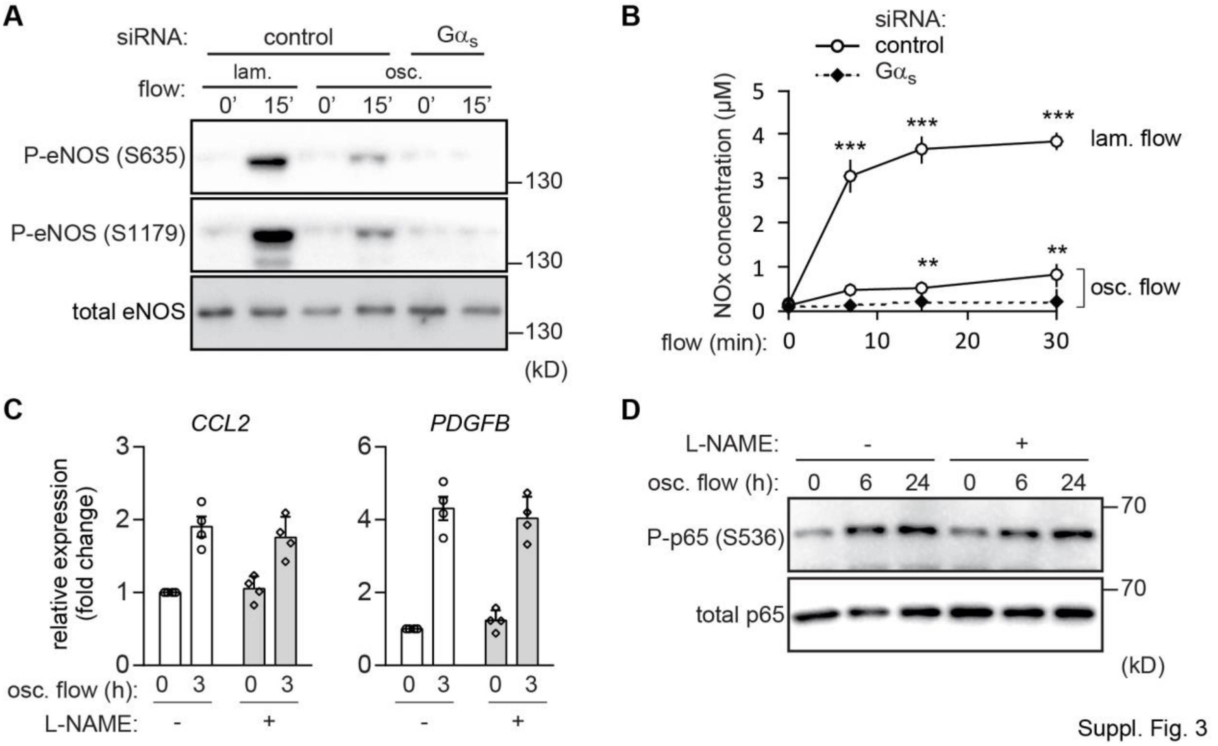

Suppl. Figure 3. eNOS activation and p65 phosphorylation induced by oscillatory flow. (A and B) Control or Gαs knock-down BAECs were exposed to oscillatory (osc.) flow or laminar (lam.) flow for the indicated time periods. eNOS phosphorylation (A) or Nitrate/nitritc levels (NOx) in the cell culture medium (B, n=3 independent experiments) were determined. **(C and D)** Confluent BAECs were incubated without or with L-NAME (100 µM) for 30 min, followed by induction of osc. flow. Inflammatory gene expression (C, n=4 independent experiments) and NF-κB (p65) phosphorylation (D) were determined. (Data represent mean values ± SD; *, P ≤ 0.05; **, P ≤ 0.01; ***, P ≤ 0.001 (two-way ANOVA and Bonferroni's post hoc test)).

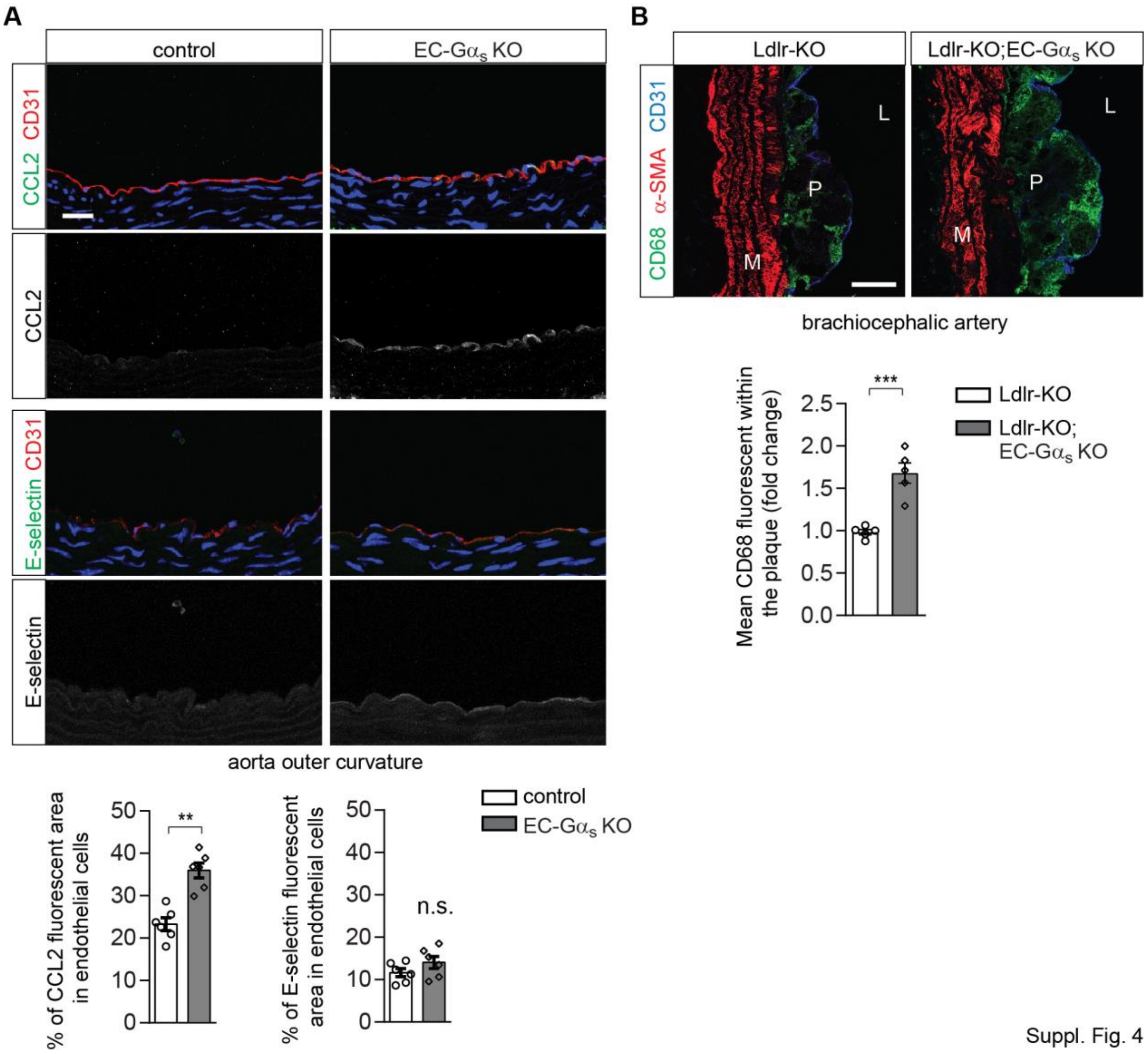

Suppl. Figure 4. Inflammatory changes in mice lacking endothelial Gαs. (A) Cross sections of the outer curvatures of aortic arches of control or EC-Gαs-KO mice stained with DAPI (blue) and antibodies against CCL2, E-selectin (green) or CD31 (red). Bar diagrams show percentage of area stained by anti-CCL2 or anti-E-selectin antibodies of total endothelial cell area stained with anti-CD31 antibody (n=6 mice per group). Bar length: 25 µm. **(B)** Immunohistochemistry of atherosclerotic plaques observed in brachiocephalic arteries using anti-CD68 antibody. Bar length: 25 µm. L: lumen, M: media wall, P: plaque. The bar diagrams show the statistical evaluation of the mean fluorescent intensity indicating CD68 within the lesion (n=5 mice per group). (Data represent mean ± SEM; **P ≤ 0.01, ***P ≤ 0.001, n.s. not significant (Student's *t*-test)).

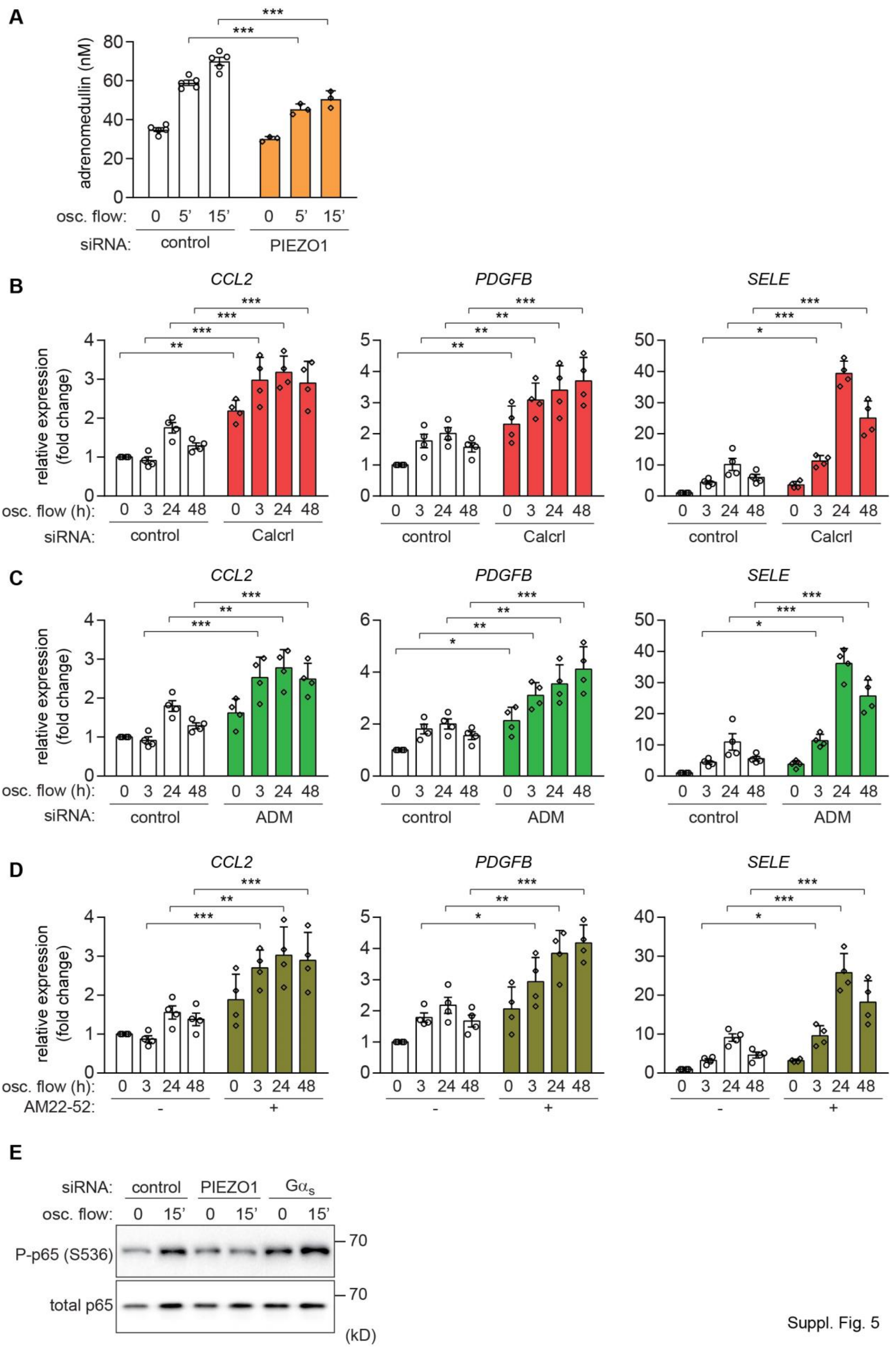
A
adrenomedullin (nM)
osc. flow: 0 5' 15' 0 5' 15'
siRNA: control PIEZO1
B
CCL2
PDGFB
SELE
relative expression (fold change)
osc. flow (h): 0 3 24 48 0 3 24 48
siRNA: control Calcrl
C
CCL2
PDGFB
SELE
relative expression (fold change)
osc. flow (h): 0 3 24 48 0 3 24 48
siRNA: control ADM
D
CCL2
PDGFB
SELE
relative expression (fold change)
osc. flow (h): 0 3 24 48 0 3 24 48
AM22-52: - +
E
siRNA: control PIEZO1 Gα$_s$
osc. flow: 0 15' 0 15' 0 15'
P-p65 (S536)
total p65
70
70
(kD)

Suppl. Fig. 5

Suppl. Figure 5. Adrenomedullin release and anti-inflammatory signaling activation in the absence CALCRL, adrenomedullin or PIEZO1. Confluent BAECs were transfected with control siRNA (control) or siRNA directed against PIEZO1 (A and E), CALCRL (B) or ADM (C), or were incubated without or with 1 µM AM22-52 (D). Thereafter, cells were exposed to oscillatory (osc.) flow. **(A)** Adrenomedullin concentration in the cell culture medium was determined (n=2 independent experiments). **(B-D)** Expression of inflammatory genes was analyzed by qRT-PCR (n=4 independent experiments). **(E)** NF-κB (p65) phosphorylation was determined by immunoblotting. (Data represent mean values ± SD; *P ≤ 0.05, **P ≤ 0.01, ***P ≤ 0.001 (two-way ANOVA and Bonferroni's post hoc test)).

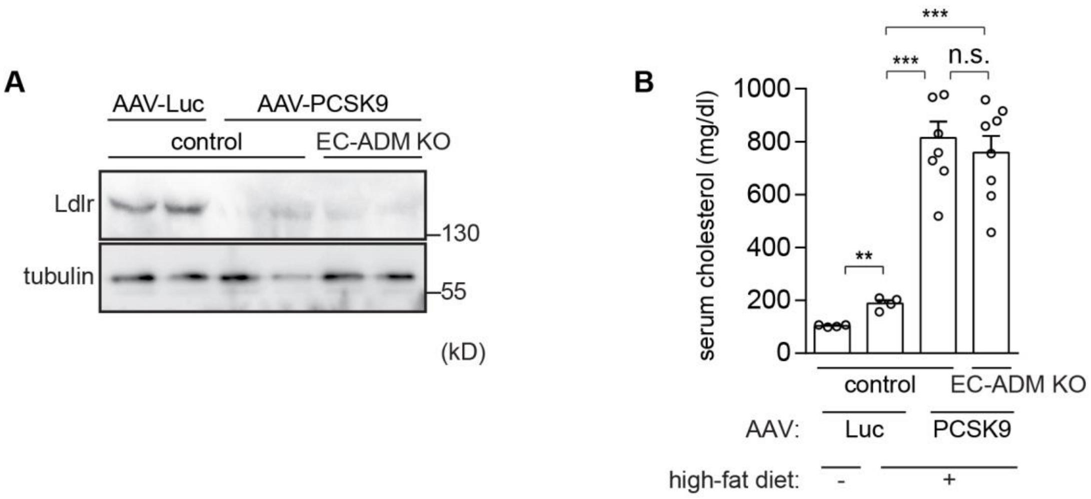

Suppl. Fig. 6

Suppl. Figure 6. Confirmation of loss of LDL receptor in the liver and induction of hypercholesterolemia by AAV-PCSK9. (A and B) control or EC-ADM KO mice were injected with AAV-PCSK9 or AAV-Luc (1 × 10^{11} VG/mouse) and fed a high-fat diet for two weeks. (A) Western blot analysis showing the expression level of LDL receptors in the liver. (B) Serum total cholesterol level from mice treated with AAV-PCSK9 or AAV-Luc and fed a normal or high-fat diet for two weeks (n=4:AAV-Luc, n=7:control;AAV-PCSK9, n=8:EC-ADM KO;AAV-PCSK9). (Data represent mean ± SEM; **P ≤ 0.01, ***P ≤ 0.001, n.s. not significant (Student's *t*-test)).

Suppl. Table 1. Effect of knock-down of different GPCRs on oscillatory flow induced NF-κB activation.

siRNA	no flow	osc. flow	ratio osc. flow / no flow
**control**	1	1,013894086	**1,013894086**
**Adora2b**	0,966292711	0,672845579	**0,697216345**
**Eltd1**	0,821819439	0,654394979	**0,80185291**
**Gpr124**	1,034879706	1,062897	**1,026439299**
**Lpar6**	0,959544177	1,068188077	**1,112072415**
**Gpr126**	1,011552949	1,133938333	**1,133374923**
**Lphn2**	0,990893907	1,121887483	**1,139250019**
**Gpr107**	1,039058363	1,190368336	**1,141775022**
**Gpr116**	1,015965376	1,158461136	**1,150643377**
**Ptger4**	0,996926447	1,164555219	**1,169831204**
**Ccrl1**	1,038192249	1,219009794	**1,174433434**
**Tpra1**	1,036796225	1,288150279	**1,249416684**
**Gpr161**	0,953154222	1,323616831	**1,380241228**
**Gpr137**	0,985451837	1,603836068	**1,60840146**
**Cxcr7**	0,981254864	1,931748948	**1,993668987**
**Calcrl**	1,170422337	2,500507013	**2,128897187**
**Gpr146**	1,194921379	3,005843384	**2,515383099**
**Gαs**	1,172386067	3,28698971	**2,802688848**

Confluent BAECs were transfected with control siRNA or siRNAs against Gα_S or the indicated GPCRs and were then exposed to oscillatory (osc.) flow for 15 min or were left unflowed (no flow). NF-κB activation was analyzed by determining p65 phosphorylation using immunoblotting as shown in Fig. 4A. Immunoblot signals were quantified and the P-p65 signal was normalized to the total p65 signal. Shown are relative values (control, no flow : 1) (n=3 independent experiments).
